# Recombination in Enteroviruses, a Multi-Step Modular Evolutionary Process

**DOI:** 10.3390/v11090859

**Published:** 2019-09-14

**Authors:** Claire Muslin, Alice Mac Kain, Maël Bessaud, Bruno Blondel, Francis Delpeyroux

**Affiliations:** 1 One Health Research Group, Faculty of Health Sciences, Universidad de las Américas, Quito EC170125, Pichincha, Ecuador; 2 Institut Pasteur, Viral Populations and Pathogenesis Unit, CNRS UMR 3569, 75015 Paris, France; alice.mac-kain@pasteur.fr (A.M.K.); mael.bessaud@pasteur.fr (M.B.); 3 Institut Pasteur, Biology of Enteric Viruses Unit, 75015 Paris, France; bruno.blondel@pasteur.fr (B.B.); francis.delpeyroux@pasteur.fr (F.D.); 4 INSERM U994, Institut National de la Santé et de la Recherche Médicale, 75015 Paris, France

**Keywords:** RNA virus, recombination, emergence, enterovirus, viral evolution

## Abstract

RNA recombination is a major driving force in the evolution and genetic architecture shaping of enteroviruses. In particular, intertypic recombination is implicated in the emergence of most pathogenic circulating vaccine-derived polioviruses, which have caused numerous outbreaks of paralytic poliomyelitis worldwide. Recent experimental studies that relied on recombination cellular systems mimicking natural genetic exchanges between enteroviruses provided new insights into the molecular mechanisms of enterovirus recombination and enabled to define a new model of genetic plasticity for enteroviruses. Homologous intertypic recombinant enteroviruses that were observed in nature would be the final products of a multi-step process, during which precursor nonhomologous recombinant genomes are generated through an initial inter-genomic RNA recombination event and can then evolve into a diversity of fitter homologous recombinant genomes over subsequent intra-genomic rearrangements. Moreover, these experimental studies demonstrated that the enterovirus genome could be defined as a combination of genomic modules that can be preferentially exchanged through recombination, and enabled defining the boundaries of these recombination modules. These results provided the first experimental evidence supporting the theoretical model of enterovirus modular evolution previously elaborated from phylogenetic studies of circulating enterovirus strains. This review summarizes our current knowledge regarding the mechanisms of recombination in enteroviruses and presents a new evolutionary process that may apply to other RNA viruses.

## 1. Introduction

Enteroviruses (EVs) constitute a large genus of small RNA viruses within the *Picornaviridae* family. This viral family represents one of the widest groups of human and animal viruses and it contains several mammal pathogens, like hepatitis A virus, foot and mouth disease virus, rhinoviruses (RVs), and poliovirus (PV), the etiological agent of poliomyelitis and prototype of EVs. Among the 15 species constituting the *Enterovirus* genus, seven contain human viruses: *Enterovirus A* to *D* (EV-A to -D) and *Rhinovirus A* to *C* (RV-A to C) [1].

Infections with human EVs are very common. They most frequently occur in children under the age of 10 and are most often asymptomatic [2]. EVs are characterized by a great phenotypic variability. Over 20 clinically recognized syndromes have been frequently associated with human EVs. Among the most frequent are pathologies of the central nervous system (CNS): poliomyelitis, encephalitis, and meningitis [3,4,5,6]. More than 90% of viral meningitis cases are caused by EVs. Enteroviral encephalitis and myelitis are less common, but more often have severe manifestations. Following the elimination of wild type PV in most regions of the world, EV-A type 71 (EV-A71) emerged as the most significant neurotropic EV. EVs are also frequently associated with acute pericarditis and myocarditis, hand-foot-and-mouth disease, pleurodynia, or respiratory disease [7,8,9,10,11]. RVs represent the principal cause of the common cold, a frequent infection both in children and adults, usually limited to the upper respiratory airways [8,10]. The three types of PV belong to the EV-C species, which also includes many weakly or non-pathogenic coxsackieviruses A (CV-A), such as CV-A13 or CV-A17.

**Figure 1:**
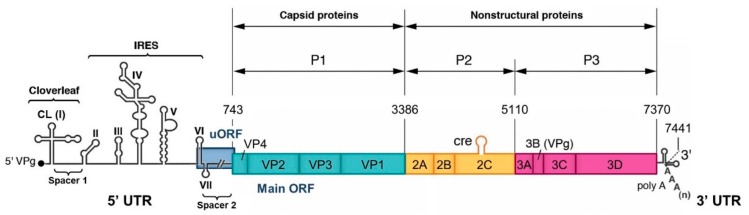
Organization of the genome of poliovirus type 1 (PV1) Mahoney. The poly-adenylated single positive-strand RNA genome is covalently linked to the viral protein VPg (also named 3B) at the 5′ terminus. In addition to the main large open-reading frame (ORF), the majority of the EV-A, EV-B and EV-C genomes, and in particular PV1 genome, contain a second upstream overlapping ORF (uORF). However, PV2 and PV3 genomes do not contain an intact uORF. The coding region is flanked by two untranslated regions (5′ and 3′ UTRs). The 5′ UTR (nucleotides 1 to 743) is magnified to indicate the seven stem-loop structures (I to VII) forming two functional units, the cloverleaf (CL: I) and the internal ribosome entry site (IRES: II-VI). The P1 region encodes the capsid proteins (VP1-4) and the P2 and P3 regions encode the non-structural proteins such as the RNA-dependent RNA polymerase 3D.

EVs are small non-enveloped viruses containing a single positive-strand RNA genome of approximately 7.5 kb in length. This genome encodes a large open reading frame (ORF) that is translated into a polyprotein processed by viral proteases (2A, 3C, and 3CD) to yield four capsid proteins (VP1-4) and non-structural proteins, such as proteases and the RNA-dependent RNA polymerase (RdRp) 3D, which are involved in the viral multiplication and the control of the cellular environment (Figure 1). Viral replication cycles entirely occur in the cytoplasm of infected cells [12]. It was recently demonstrated that the majority of EV-A and EV-B genomes and around half the EV-C genomes contain a second ORF located upstream and overlapping the polyprotein ORF (ppORF) [13]. This second upstream ORF (uORF) encodes a single protein that may play a role in virus growth in gut epithelial cells, which are the entry site of these viruses into a susceptible host. Two untranslated regions flank the coding region of EV genome (5′ and 3′ UTR). The EV 5′ UTR is about 740 nucleotides in length and it contains seven highly conserved stem-loop domains (I to VII) forming two functional units (Figure 1). Domain I forms a cloverleaf (CL) structure that is required for initiating both negative- and positive-strand RNA synthesis [14,15,16,17]. Domains II to VI (dII to dVI) contain the internal ribosome entry site (IRES) that initiates cap-independent translation by interacting with canonical and noncanonical cellular translation factors to recruit ribosomes [18,19]. The CL and IRES elements are separated by a short pyrimidine-rich sequence, named spacer 1, and the IRES is linked to the initiation AUG codon of the ppORF by dVII and a poorly structured sequence of about 100 nucleotides, named spacer 2 [20]. This dVII-spacer 2 region contains the main part of the uORF that is present in many EV genomes [13].

EV transmission is generally by fecal-oral contamination or by respiratory droplets. In the case of the EVs transmitted by the fecal-oral route, such as PV, viral particles infect the oropharyngeal and intestinal mucosa. The virus efficiently multiplies in the intestine and it is excreted in stools for several weeks. From the digestive tract, the EV reaches cervical and mesenteric lymph nodes and then establishes a primary viremia. The infection usually comes to an end at this stage, and therefore most of EV infections are asymptomatic. However, the virus might reach other tissues and organs, depending on his tropism, which results in a secondary viremia and the possible development of syndromes [2]. PV invades the CNS, its target organ, in less than 1% of infection. The virus specifically infects and destroys motor neurons, inducing the irreversible flaccid paralyses that are typical of poliomyelitis [21].

**Figure 2:**
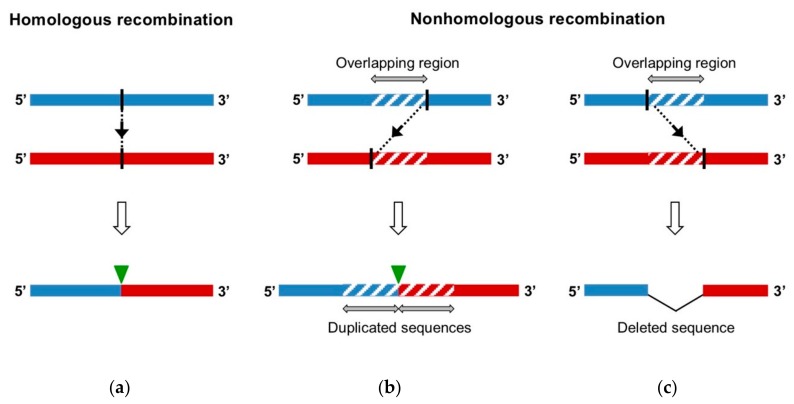
Homologous and nonhomologous recombinant genomes. Parental RNA genomes are located in the upper panel of (**a**), (**b**) and (**c**) diagrams. They have a similar genomic structure. The recombination site in each of the parental genomes is represented by a black vertical line, the recombination event is indicated by black dotted line and arrow. The recombination site in the obtained recombinant genome (lower panels) of (**a**) and (**b**) diagrams is indicated by a green reversed triangle. (**a**) Homologous recombination occurs at the same site in both parental genomes, thus the obtained recombinant has the same genomic structure as the parental viruses. (**b**) and (**c**) Nonhomologous recombination occurs at different sites in the two parental genomes. (**b**) a duplication of homologous sequences (hatched) is generated around the recombination site. (**c**) A deletion of genomic sequence is generated.

As RNA viruses, EVs are characterized by a great genetic variability relying on two different evolutionary mechanisms: mutation and recombination. Firstly, the lack of proofreading activity of the 3D polymerase leads to a high mutation rate and the generation of a population of related sequences, named quasispecies [22]. The extent of this mutant swarm is crucial for viral adaptability, dissemination, and pathogenesis [23,24,25,26,27]. The other major driving force in RNA virus evolution is genomic RNA recombination. RNA recombination is a molecular process, during which genomic fragments that belong to distinct RNA strands are combined in a single genome. Recombination is classified regarding the features of the recombination site of the produced genome, without prior knowledge of the underlying generation mechanism (Figure 2) [28]:

- Homologous recombination occurs at the same site in both parental genomes, therefore no insertion or deletion is observed at the recombination site when the recombinant genome is aligned with parental genomes.
- Nonhomologous recombination occurs at different sites in the two involved genetic fragments, generating aberrant structures, such as deletions or duplications of homologous parental sequences on each side of the recombination site.

Moreover, depending on the origin of the parental strands, we can distinguish between intra-genomic recombination, or rearrangement, wherein the recombining strands belong to the same molecule, and inter-genomic recombination, wherein the fragments have different origins [29].

RNA recombination allows for the exchange of genetic information and incorporates viral RNA fragments into new genomic contexts. Thus, it favors both the combination of beneficial mutations into the same genome, leading to the creation of variants that are best adapted to withstand environmental selective pressure, and the elimination of negative combinations of mutants from the population [30]. RNA recombination enables viruses to quickly explore a greater proportion of the sequence space than is accessible by point mutations [31]. In addition to being a source of genetic diversity, RNA recombination has been shown to be a repair mechanism, essential to maintain viral genome integrity [32,33,34]. Recent studies in PV demonstrated that effective RNA recombination is critical to rapid adaptation to dynamic selective environments and that, in the infected host, the concerted activities of mutation and recombination are required to overcome tissue-type specific antiviral selection and to establish robust infection and virulence [35,36].

As an adaptive mechanism, RNA recombination has played an important role in the diversification and evolution of RNA viruses, and resulted in new isolates, lineages, species, or even new families [37,38,39,40,41,42,43]. Naturally occurring recombination events between viruses from different families that led to the transfer of a functional RNA element or gene sequence and then resulted in a gain of function have also been described [44,45,46]. Moreover, RNA recombination has been associated with cross-species transmission and the expansion of viral host range [47,48,49,50,51]. Finally, RNA recombination can lead to an increase of viral pathogenicity [52,53,54] and fitness [55,56,57,58].

In particular, RNA recombination is thought to contribute to the emergence of pathogenic circulating vaccine-derived PVs (cVDPVs) that have been complicating the World Health Organization program for the global eradication of poliomyelitis. This eradication program, which was launched in 1988 and has been largely successful, mainly involved massive vaccination campaigns with the oral polio vaccine (OPV), which is composed of live attenuated strains of the three PV types, Sabin 1, 2, and 3. These strains are only able to replicate to high titers in the digestive tract, conferring strong systemic and intestinal immunity that limits subsequent PV replication and viral transmission among humans [59]. However, the OPV strains are inherently genetically unstable and suboptimal vaccine coverage may allow for their circulation among humans not adequately immunized with OPV, which leads to genetic drift and the emergence of new pathogenic strains, known as cVDPVs [60,61,62,63]. Since 2000, cVDPVs have caused nearly 30 poliomyelitis outbreaks worldwide [64]. Most cVDPVs studied to date have recombinant genomes, composed of sequences that are derived from the OPV strain, with more than 1% nucleotide substitution, for at least the region encoding capsid proteins and sequences originating from other EV-Cs, especially from CV-A13 and CV-A17 types, for some or all of the rest of the genome, in particular the region encoding non-structural proteins [61,65,66,67,68,69,70,71,72,73]. By facilitating the replacement of attenuating vaccine sequences in a single event, RNA recombination was found to influence the phenotypic characteristics of the cVDPVs, including their fitness and pathogenicity [74,75,76,77,78].

This review first focuses on the studies of circulating EV strains that led to the elaboration of a theoretical model of EV evolution that is based on highly frequent recombination involving exchanges of functional genetic modules. Subsequently, after describing the two mechanisms of RNA recombination, we present the systems that have been recently used to experimentally study intra- and intertypic EV recombination. The experimental results suggest, firstly, that EV recombination is a multi-step process and, secondly, that it occurs at preferential sites along the genome, thus supporting the theoretical model of EV evolution through modular intertypic recombination.

## 2. Theoretical Model of Enterovirus Evolution Through Modular Intertypic Recombination

### 2.1. High Recombination Frequency in Enteroviruses

RNA recombination appears to occur extremely frequently in EVs. Recombination frequencies of 10^−6^ per site per generation were estimated during co-infection experiments [74,79]. In the EV-infected population, the recombinant forms are regularly identified, resulting from genetic exchanges between viral strains within the same type, between different types within the same species, or even between different species in the case of recombination occurring in the 5′ UTR [80,81,82,83,84,85,86,87,88,89,90]. This high naturally-occurring recombination frequency is partly due to the fact that EVs display several ecological and biological features that were identified as being necessary for the occurrence of genetic exchanges and the emergence of viable recombinant viruses [28,91]:

- Co-circulation: Several studies evaluating the circulation and genetic diversity of EVs in restricted geographic areas and on a short period of time revealed an extensive co-circulation of a high number of types from the four human EV species, usually associated with a high intra- and intertypic recombination frequency [65,92,93,94,95,96,97].Host co-infection: Consistent with the intense co-circulation observed, many cases of multiple infections in individuals have been reported [65,80,98].
- Cell co-infection: It was recently demonstrated that PV can spread as one unit containing multiple viral particles, either within lipid vesicles or as viral aggregates, and this delivery mode increased coinfection frequency and infectivity [99,100]. Furthermore, another recent study showed that certain resident bacteria of the gastrointestinal tract bind PV, increase viral co-infection of mammalian cells and enhance viral recombination, even when the ratio of virus to host cells is low, such as during the first cycle of replication following inter-host transmission [58].
- Colocalization of parental genomes: As all positive-strand RNA viruses, EVs replicate their genomes in virus-induced, membrane-bound replication compartments. The study of cells co-infected with two different PV strains showed that the majority of replication complexes contained both viral genomes, early in infection [101]. Moreover, a recent study in Brome mosaic virus (BMV) showed that the structure and size of the virus membranous replication compartments play a fundamental role on recruitment of multiple RNAs into a contiguous space, and thus on inter-genomic RNA recombination frequency, and accordingly suggested that the PV replication structures might favor RNA recombination [102,103].
- Selection: Finally, generated recombinant genomes have to be viable and able to efficiently compete with parental genomes and to confront bottleneck events occurring during virus life cycle, to spread in the viral population. This implies a structural and functional compatibility of the different recombining sequences, as well as a certain tolerance to genomic alterations in order to limit their negative consequences.

### 2.2. Analysis of Recombination Events in Circulating Enterovirus Strains

The first phylogenetic analysis of complete genomes of EV prototype strains highlighted the significant role of recombination in the evolution of the human EV species [104]. The results of intraspecies comparisons by bootstrap and genetic similarity analyses provided strong evidence that multiple homologous recombination events, both within and between types, which led to the shuffling of genomic fragments between various strains, had shaped the evolution of each EV species. Moreover, the study suggested that two early recombination events at the junction of the 5’ UTR and coding region of the species progenitors could explain that current human EV 5’ UTR sequences cluster into two distinct major phylogenetic groups: group I, which is comprised of EV-C and EV-D, and group II, which is formed by EV-A and EV-B. This work was the first one to suggest the concept of independent evolution of different genome fragments. Thereafter, these results were confirmed by several studies that analyzed and compared the phylogenetic relationships in different genomic regions of prototype and field strains [80,81,83,84,85,87,105,106,107,108]. Within each human EV species, radical incongruent tree topologies between the untranslated, structural and non-structural regions, and even between different proteins across the non-structural region of the genome, indicated frequent intra- and intertypic recombination during the evolution of EV types. Such studies provided evidence that RNA recombination had played an important role for EV speciation and it remains a major driving force in the ongoing evolution of EVs within each species.

Full-genome comparisons and sequence similarity analyses of prototype and circulating EV strains led to the suggestion that genetic restrictions might influence the recombination location and frequency [86,104,109,110,111]. Firstly, the junctions between the 5’ UTR and the structural region and between the structural and non-structural regions were identified as putative recombination hotspots, flanking a structural region where recombination was virtually absent. Indeed, although EVs displaying chimeric capsid proteins have been documented [112,113,114,115], intertypic recombination events appeared to occur almost entirely outside of the capsid-encoding region [86,107,108,116], which suggests that it is a relatively stable unit. This could be explained by structural incompatibilities between capsid proteins from different types during virus assembly or maturation, or during receptor binding, when the parental viruses use different receptors [107,111]. By contrast, multiple studies provided evidence of the occurrence of extensive series of recombination events throughout the entire non-structural region, which seemed to be relatively randomly distributed and resulted in complex mosaics of sequences [86,104,108,109,110,111,117]. These observations suggest that each non-structural protein might be functionally interchangeable with any other variant within each species. Secondly, recombination appears to only occur among members of a given species. The frequency and dynamics of recombination seemed to be different between the human EV species [109,111]. For example, time-correlated recombination events might be more frequent in EV-B than in other human EV species [111]. Moreover, within each human EV species, type-specific recombination frequencies have been observed, with a few types functioning as preferential recombination partners [84,86,108,118]. Subgroups within a given species could also be defined, depending on recombination dynamics [119].

### 2.3. Genetic Features of Recombinant Circulating Vaccine-Derived Poliovirus Genomes

Most of the cVDPVs described so far have mosaic genomes that are composed of mutated OPV sequences and sequences related to non-PV EV-Cs [61]. The analysis of the cVDPV strains implicated in outbreaks of poliomyelitis in Cambodia [120] and Madagascar [65,70] revealed that the non-PV EV-C sequences that are present in the genomes of these strains were related to co-circulating non-pathogenic CV-A strains, especially from CV-A13 and CV-A17 types. Several closely related EV-C types are actually thought to be able to function as recombination partners for OPV strains [108]. Thus, the multiplication and circulation of OPV strains in close interaction with other EV-Cs within a diverse EV ecosystem led to exchanges of genetic fragments through intertypic recombination and the emergence of these cVDPV strains [65,70,86].

**Figure 3:**
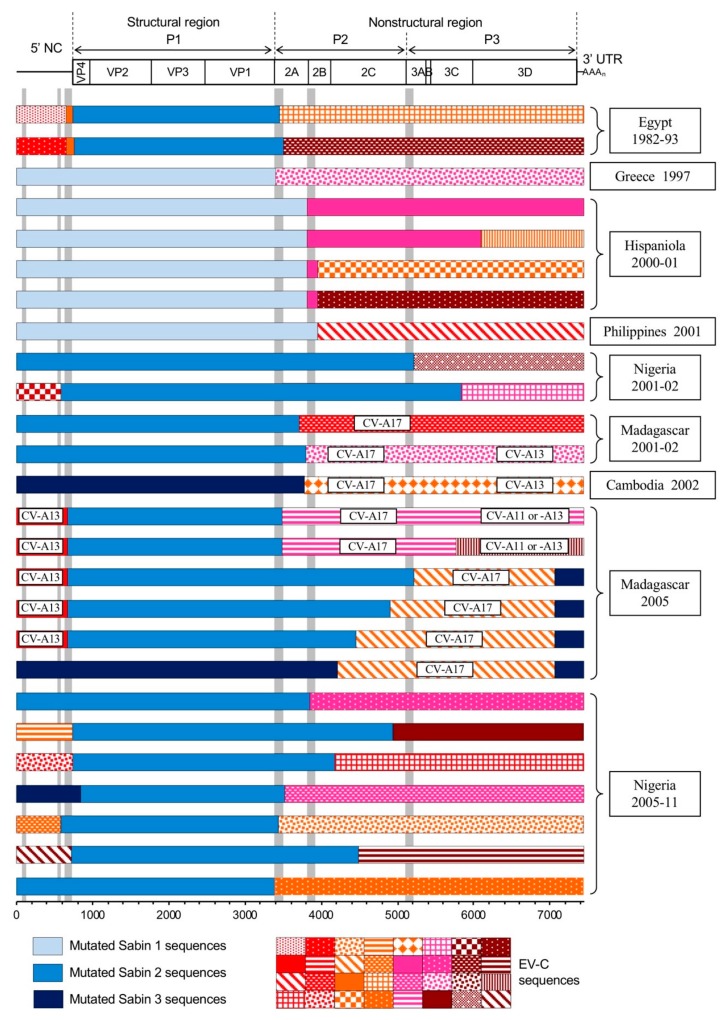
Genomic structures of all recombinant circulating vaccine-derived poliovirus lineages characterized so far. A schematic view of the genetic organization of the poliovirus genomes is given in the upper panel (see also Figure 1). The presence of vaccine-derived sequences is indicated (mutated Sabin 1, 2, of 3 sequences) as well as the non-vaccine sequences derived from other species C enteroviruses (EV-Cs). Colors and patterns differentiate EV-C sequences that differed significantly from each other. Non-vaccine sequences showing similarity with those of co-circulating coxsackieviruses A (CV-A11, -A13, -A17) are indicated. Data are modified from [73] (Egypt), [67] (Greece), [68] (Hispaniola), [69] (Philippines), [71,72] (Nigeria), [65,66,70,76] (Madagascar), [120] (Cambodia). The location of the six recombinant hotspots identified by experimental studies of genetic exchanges between poliovirus and enteroviruses is indicated by grey rectangles [77,121,122] (see further in the text).

Moreover, most of the recombinant cVDPV genomes studied to date displayed similar genetic patterns. They were homologous recombinants that had kept at least the entire structural region of OPV strains, with more than 1% nucleotide substitutions, and some or all of the 3’ half of the genome was derived from non-PV EV-C sequences. In most of them, the recombination sites were located in proteins 2A or 2B (Figure 3) [65,67,68,69,73,120]. Nevertheless, recombination sites could also be found elsewhere in the non-structural region [66,70,71]. In addition, many VDPV lineages also displayed a 5’ UTR that was acquired by intertypic recombination [66,70,71,72,73]. Thus, most of the cVDPV genomes resulted from the association of several genetic segments from different phylogenetic origins. cVDPV genomic structures could be highly complex, as some cVDPV lineages showed quadripartite recombinant genomes between OPV and non-PV EV-C sequences [70]. The location of the recombination sites, delimiting the genetic segments, matched the preferential recombination regions that were identified by the phylogenetic studies presented previously: the two extremities of the structural region and the entire non-structural region (Figure 3).

### 2.4. Modular Intertypic Recombination Hypothesis

Genomic and phylogenetic analyses of EV strains from the four human EV species, including cVDPVs, highlighted the essential role of recombination in the evolution and the genetic architecture shaping of EVs. It became clear that the type classification that is used for EVs and based on the degree of similarity between the strains in the region encoding capsid proteins is generally not reflected in the non-structural region and the 5’ UTR [104]. EV genome seems to be constituted of different genetic fragments that follow different evolutionary paths. Indeed, while the range of EV types circulating in human population, often episodically, remains relatively constant, the accumulated set of distinct genetic lineages of 3D polymerase encoding sequences is far greater and their occurrence far more transitory [123]. The location of the putative recombination sites, delimiting the genomic fragments, suggest that the 5’ UTR and the capsid-encoding region could both be considered as recombination units within which recombination is probably constrained by genetic and/or structural requirements, unlike the non-structural region, where each protein-coding sequence could be exchanged as a single unit [107,108].

These observations led to the elaboration of a theoretical model of EV genetics. EV types cannot be considered as “subspecies” with independent evolutionary patterns, but rather EV species would consist of a finite set of capsid genes that are responsible for different types and a swarm of non-structural protein genes and untranslated regions [83,106,110]. These different genomic fragments would evolve independently and combine freely and frequently through RNA recombination during co-infections, potentially producing variants with new phenotypic properties. This mode of evolution through modular recombination would provide a high level of flexibility and a capacity for very quick evolutionary changes to the EVs, and could be viewed in particular as an adaptative response to the immune system of their hosts [106].

## 3. Two Main Mechanisms of RNA Recombination in Enteroviruses

**Figure 4:**
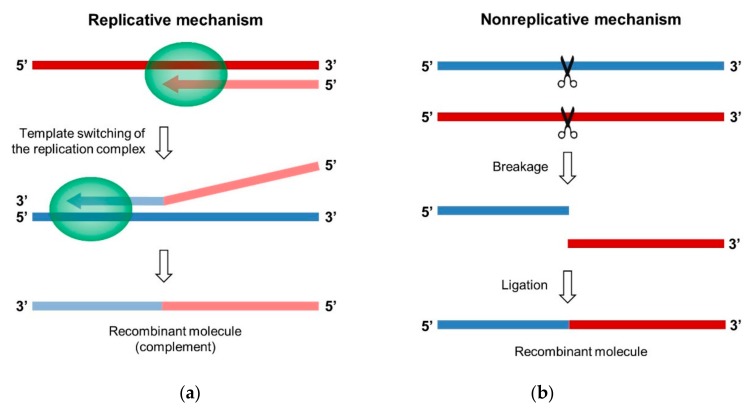
Mechanisms of replicative and nonreplicative RNA recombination. (**a**) The replicative mechanism of copy-choice. The replication complex pauses during the synthesis of the complementary strand of the RNA donor (in red) and dissociates from the RNA donor template. Then, the incomplete nascent RNA strand interacts with the acceptor RNA (in blue) where the replication complex reassembles and the synthesis of the complementary strand resumes. The complementary strand is indicated by lighter colors. (**b**) The nonreplicative mechanism of breakage-ligation. The two parental RNA molecules are degraded, and then the two fragments generated are covalently linked.

Two different mechanisms can lead to the generation of a recombinant RNA molecule in RNA viruses, including EVs: the replicative “copy-choice” mechanism and the nonreplicative “breakage-ligation” mechanism (Figure 4). Both were first described in PV [79,124].

### 3.1. The Replicative Mechanism of Copy-Choice

The copy-choice has been demonstrated in many RNA viruses, including retroviruses, and it is considered as the major viral RNA recombination mechanism [30,125,126].

#### 3.1.1. Template Switching of the Viral Polymerase

The model that was proposed for the copy-choice mechanism postulates that the neo-synthesized nucleic acid chain can dissociate from the RNA donor template during replication and interact with a different template, the acceptor RNA, or with a different region of the same template. This interaction results in the transfer of the replication complex to the new template, where RNA synthesis resumes, which produces a hybrid genomic molecule that contains genetic information from two different sources (Figure 4a) [28,79]. Template switching can produce homologous, as well as nonhomologous, recombinants.

The first experimental evidence supporting the copy-choice mechanism was provided in 1986 by Kirkegaard and Baltimore, despite being initially proposed by Cooper et al. as a model of recombination in PV [127], who demonstrated that RNA synthesis was necessary for PV recombination [79]. Following this study, the replicative copy-choice model could be confirmed and generalized to other RNA viruses and retroviruses, and many studies tried to characterize the details of its molecular mechanism [125,128,129]. The template switching capacity of the PV 3D polymerase was demonstrated while using purified reconstituted in vitro systems [130]. The possibility for the 3D polymerase to use the 3’ end of the incomplete nascent RNA strand as a primer to be elongated on a new template was also confirmed [131,132,133]. The identification of mutations in the 3D polymerase that negatively or positively affect recombination frequency further supported the replicative recombination mechanism [35,121,134,135].

Nevertheless, the exact molecular mechanism of the copy-choice remains to be elucidated. For that matter, different mechanisms may exist according to the features of the viral polymerase and replication complex, or else, several mechanisms might be possible for a single polymerase. Distinct possible mechanisms have been postulated [125,135,136]: (i) the elongation complex dissociates from the RNA donor template and the polymerase—incomplete nascent RNA strand complex interacts with a new template, the acceptor RNA; (ii) the incomplete nascent RNA strand alone dissociates from the elongation complex, interacts with the acceptor RNA, and recruits a new polymerase; (iii) the incomplete nascent RNA strand dissociates, leaving the polymerase—RNA donor template complex that associates with a new incomplete nascent strand; and, (iv) the RNA donor and acceptor templates closely hybridize and template switching occurs without dissociation of the elongation complex. In the case of PV, recent findings by Kempf et al. on the structure of the 3D polymerase and the elongation complex seem to favor the second possibility mentioned above [135]. The identification of regions that are capable of forming stable heteroduplexes at the vicinity of certain recombination sites in PV genome suggested that the fourth mechanism mentioned above could also be implicated in some cases [125,137].

Finally, for some positive-strand RNA viruses, including PV, it was postulated that template switching preferentially occurs during negative-strand RNA synthesis [79,138,139], whereas, for other viruses, it would either preferentially occur during positive-strand RNA synthesis [140,141] or indifferently between either strand [142,143].

#### 3.1.2. Factors Influencing Template Switching

Many factors are supposed to favor template switching, by having an effect on the pausing of the synthesis on the donor strand, which destabilizes the replication complex and promotes its dissociation, or by playing a role in the association of the nascent strand and the polymerase to the acceptor RNA.

The high replication speed of RdRps, as optimized by natural selection, results in a high incorporation of incorrect nucleoside triphosphates (NTPs), which is associated with an increased number of pause events that may favor elongation complex dissociation [144,145,146,147]. Stable secondary, or even tertiary, structural features in the donor RNA molecule were described in many RNA viruses as a critical factor for the slowing and destabilization of the elongation complex [148,149,150,151]. In particular, secondary structures were identified as recombination hotspots in PV genome [152,153,154]. Various other factors were found to promote template switching, including heteroduplex formation between parental RNAs [139,155,156,157,158], the presence of breaks in the RNA template [128,149], and low NTP concentrations [159]. Finally, recombination sites were often found to be associated with AU-rich sequences in several positive-strand RNA viruses, including PV [30,139,160,161,162,163]. The weak annealing of A-U nucleotides is supposed to facilitate the dissociation of the nascent strand from the complementary donor strand inside the replication complex, and thus the template switching initiation.

After dissociation from the donor template, the replication complex and the nascent strand need to bind the acceptor template to re-initiate nucleic acid synthesis. The first feature identified as a factor that affects recombination frequency and location is the sequence identity level between the nascent strand and the acceptor RNA [125,129]. Indeed, many studies in PV and other positive-strand RNA viruses showed a direct correlation between the degree of sequence identity of the templates and the recombination frequency [79,121,164,165]. The sequence identity level between the nascent and acceptor strands is also thought to promote homologous recombination, by enabling the two strands to extensively dimerize and induce a precise strand switch [28]. Moreover, in the case of PV and BMV, GC-rich sequences could be associated with an increase of recombination frequency in the vicinity of these sequences and are thought to promote the annealing of the incomplete nascent RNA strand to the acceptor RNA template [153,161]. This interpretation suggests that, in these viruses, thermodynamic factors influence the annealing of the nascent strand to the acceptor RNA to a greater extent than the initial dissociation from the donor template, which is conversely hampered by the strong annealing of G-C nucleotides [153].

Host and environmental factors are also supposed to be implicated in the copy-choice mechanism. Studies in tombusvirus and hypovirus led to the identification of various cellular pathways and factors that are involved in viral replicative RNA recombination [166,167,168,169,170].

### 3.2. The Nonreplicative Mechanism of Breakage-Ligation

#### 3.2.1. Demonstration in Poliovirus

An alternative recombination model that is fundamentally different from the copy-choice was first advanced on the basis of experimental data obtained in a cell-free system that employed purified bacteriophage Qβ replicase to detect replicable RNAs that were generated from nonreplicable RNA fragments [171,172]. However, the presence of Qβ replicase required for amplification of the recombinant molecules did not fully exclude a replicative mechanism.

Following these studies, the existence of a recombination mechanism not involving the viral polymerase was unambiguously demonstrated in vivo in PV while using pairs of defective complementary genomic RNA fragments [124,136,173]. The transcript containing the functional 5’ part of the viral genome is called 5’ partner, the one providing the functional 3’ part is the 3’ partner. In a first pair configuration, the 5’ partner comprised the entire 5’ UTR only and the 3’ partner was made from the complete PV genome, in which the IRES was mutated or deleted [124]. Co-transfecting cells with these two complementary genomic RNA fragments that are unable to be translated or to replicate led to the production of infectious genomes that are recombinant in the dVII-spacer 2 region linking the 5’ UTR to the ppORF. Homologous as well as nonhomologous recombinant genomes were isolated in this highly permissive genomic region. These results suggested that RNA recombination could occur in the absence of a functional RdRp.

This hypothesis was confirmed even more rigorously with another pair configuration, in which the two RNA fragments corresponded to the PV genome with a break in the RdRp-coding region [173]. Since each fragment only contained a part of the viral RdRp gene, this enzyme could not be involved in the first steps of the generation of the recombinant molecule. In the case of RNA partners supplementing each other precisely, a single ligation by phosphodiester bond would restore the integrity of the PV genome. Co-transfecting cells with this partner pair only yielded viable viruses when the 5’ partner contained a phosphorylated 3’-nucleotide (3’-P) and the 3’ partner harbored a 5’ hydroxyl group (5’-OH). In the case of overlapping RNA fragments, a majority of homologous recombinant genomes were isolated, and an association was observed between the recombination site location and the terminal nucleotide structure of both partners. When the cells were co-transfected with unmodified RNA partners, i.e., carrying 5’ triphosphate and 3’-OH ends, recombination sites were located at internal positions within the overlapping sequence. In contrast, when using a 5’ partner with a 3’-P or a 3’ partner with a 5’-OH, the activated fragment was entirely incorporated into the genome in most recombinants [173].

Subsequently, the generation of infectious recombinant genomes in the absence of functional RdRp was also demonstrated in Bovine viral diarrhea virus (BVDV) and Hepatitis C virus (HCV), from the *Flaviviridae* family, while using similar co-transfection systems with defective RNA partners, which suggests that the mechanism of nonreplicative recombination could be common to several positive-strand RNA viruses [174,175,176,177]. Whereas the replicative copy-choice model is widely admitted in all RNA viruses, there is now evidence that recombinant RNA genomes can also be produced through a fundamentally different nonreplicative mechanism. This mechanism could represent an alternative or parallel pathway to replicative recombination in vivo, at least in some positive-strand RNA viruses, including EVs.

#### 3.2.2. Putative Implication of Cellular Factors

The details of the mechanism(s) that lead to the generation of recombinant genomes in the absence of viral RdRp still remain unknown. The studies previously mentioned, performed in PV, BVDV, and HCV, suggested the existence of a common mechanism of breakage-ligation, very likely involving cellular factors [124,173,175,176,177]. Recombining RNA fragments could be generated by sporadic bond dissociation or, more probably, by cellular exo- and endoribonucleases, as suggested by the fact that recombination preferentially occurred in single-stranded regions and was promoted by fragments carrying 3’-P and 5’-OH ends [173,175]. Such RNA fragments can actually be produced in vivo, in particular by endoribonucleolytic cleavages [178]. These activated 3’-P and 5’-OH fragments would then be rejoined by cellular ligases or through self-ligation [28]. Indeed, human cell extracts have been shown to be able to ligate 3’-P-terminated RNA substrates, probably through a preliminary modification by a cyclase [179]. A positive linear correlation between RNA concentration and recombination frequency was actually observed in BVDV and HCV, as would be predicted by a random breakage-ligation mechanism [175,176]. Thus, the nonreplicative RNA recombination mechanism might resemble the process of enzymatic splicing leading to the production of mature tRNA in vertebrates [179]. Nevertheless, the cellular factors that are implicated in nonreplicative recombination have not been identified yet.

#### 3.2.3. Alternative Mechanisms of RNA Recombination not Involving Viral RNA-Dependent RNA Polymerase

Other mechanisms of RNA recombination have been proposed to explain the recovery of complete viral genomes from defective RNA fragments in the absence of viral RdRp. In particular, it was demonstrated that the RNA molecules are able to undergo spontaneous nonenzymatic intermolecular transesterification reactions [180,181,182,183]. This ability is linked to the predisposition of RNA to self-assembly, which enables it to form multi-motif functional complexes (ribozymes) where consecutive cleavage-ligation reactions can be performed. This innate capacity for nonenzymatic recombination is thought to have contributed to the development of the RNA world. This mechanism could be implicated, in particular, in the generation of recombination products containing a recombination site located within RNA secondary structures, such as pseudo-knots, bulges or loops [124].

In addition, the fact that RNA recombination can occur in the absence of a functional RdRp does not completely exclude a replicative mechanism of primer extension that would be mediated by cellular polymerases [174]. So far, cellular RdRps have not been identified in mammals. However, host DNA-dependent RNA polymerases, such as RNA polymerase II, have been reported to replicate and recombine RNA genomes of HDV and plant viroids [184,185,186]. Even though RNA replication of most RNA viruses, including picornaviruses and flaviviruses, occurs in the cytoplasm and promoter-like elements for cellular polymerases have not been described in the genomes of these viruses, a possible RNA recombination mechanism of primer extension by a host polymerase cannot be fully excluded.

## 4. Recent Experimental Systems Designed to Study Recombination in Enteroviruses

**Figure 5:**
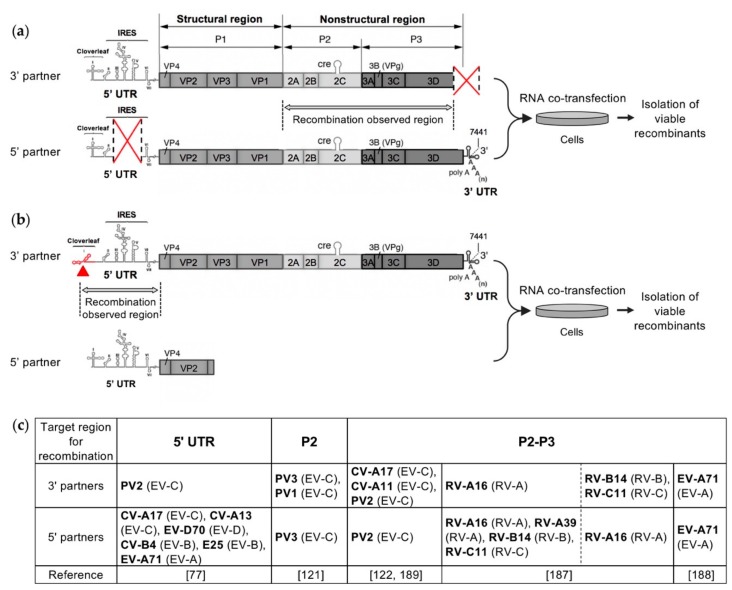
Experimental systems of intra- and intertypic recombination between enteroviruses. (**a**) and (**b**). Examples of recombination partners designed to target recombination in the P2-P3 region (**a**) and in the 5′ UTR (**b**). (**a**) The 3′ partner is made from the complete enterovirus genome in which the 3′ end of the 3D polymerase and the entire 3′ UTR were deleted. The 5′ partner is made from the enterovirus genome carrying a deletion in the IRES. Red crosses indicate the genomic regions in which deletions were made. Co-transfecting cells with these two defective complementary genomic RNA fragments led to the production of infectious genomes recombinant in the P2-P3 region [122,189]. (**b**) The 3′ partner is made defective by substitutions in the cloverleaf structure of 5′ UTR. The 5′ partner includes the complete 5′ UTR followed by the N-terminal part of the ppORF. Co-transfecting cells with this pair of defective genomes will generate viable viruses only if a recombination event occurs in the 5′ UTR [77]. (**c**) List of enterovirus types used for the construction of the 3′ and 5′ partners in the different experimental systems. For each type, the enterovirus species is indicated in brackets.

Several recent studies have investigated the intra- and intertypic EV recombination process in experimental settings in order to understand the rules governing genetic exchanges between EVs, that can lead in particular to the emergence of pathogenic recombinant cVDPVs [77,121,122,187,188]. In these studies, similar recombination cellular systems were developed mimicking natural genetic exchanges between EVs. These systems were based on the co-transfection experiments previously described and carried out by Gmyl et al. to study nonreplicative recombination in PV [124,173]. Their principle relied on the rescue by recombination of a defective EV RNA genome after co-transfecting cells with an infectious or defective complementary EV genomic RNA (Figure 5). According to the EV strains that were chosen to construct the RNA partners, the systems enabled studying intratypic, intertypic, or even interspecies recombination. Depending on the design of the two RNA partners, recombination was targeted to a specific genomic region, either the non-structural region [121,122,188] or the 5’ UTR [77], or could occur in the major part of the viral genome [187]. Following co-transfection, viable recombinant viruses were isolated as early as possible to minimize their loss or evolution through continued propagation and competition, and thus to analyze early recombination events in EVs. The genomic sequences of the recombinant viruses were then compared with those of the parental partners, in order to determine the location and structure of the recombination sites [77,121,122,187,188].

All of these experimental studies of intra- and intertypic recombination in EVs provided similar results regarding the features of the generated infectious recombinant genomes, in particular the ratio of homologous to nonhomologous recombinants and the location of recombination sites, regardless of the tested RNA partner pair and the genomic region targeted for recombination. No significant differences in the genomic structure of the obtained recombinants were observed whether the defective EV genome was rescued with a replicable or nonreplicable RNA fragment, i.e., whether the initial step in the generation of the recombinants involved a replicative or nonreplicative RNA recombination event [121,122].

## 5. The Generation of Homologous Intertypic Recombinant Enteroviruses, a Multi-Step Process

Both homologous infectious recombinant genomes and nonhomologous ones, showing deletions or insertions at the recombination site, were recovered from the recombination cellular systems previously described. When both RNA partners belonged to the same type, homologous recombinant genomes were mostly isolated [121,122]. On the contrary, intertypic and interspecies recombination generated mainly nonhomologous recombinants [77,121,122,187]. Very few, 0 to 4%, isolated genomes recombinant in the non-structural region showed deletions at the recombination site, and the length of the deleted sequence never exceeded two codons, reflecting the fact that nonhomologous recombination generating deletions of coding sequences is likely to produce non-viable or non-competitive genomes [121,122,187]. However, 33% of the genomes with a recombination site in the 5’ UTR displayed deletions, up to 164 nucleotides, in particular, when the recombination site was located in the dVII-spacer 2 region, between the IRES and the ppORF initiation codon [77]. These recombinants appeared to be very stable upon successive cellular passages and fitter than some homologous recombinants in the 5’ UTR. These observations are consistent with previous reports that suggested the dVII-spacer 2 region of EVs can tolerate profound modifications without significant phenotypic changes [190,191]. Indeed, one of the 12 natural recombinant type 2 cVDPV lineages with non-PV EV 5′ UTR sequences that have been described so far showed a shorter spacer suggesting that a deletion occurred during recombination [70,71,72,73].

**Figure 6:**
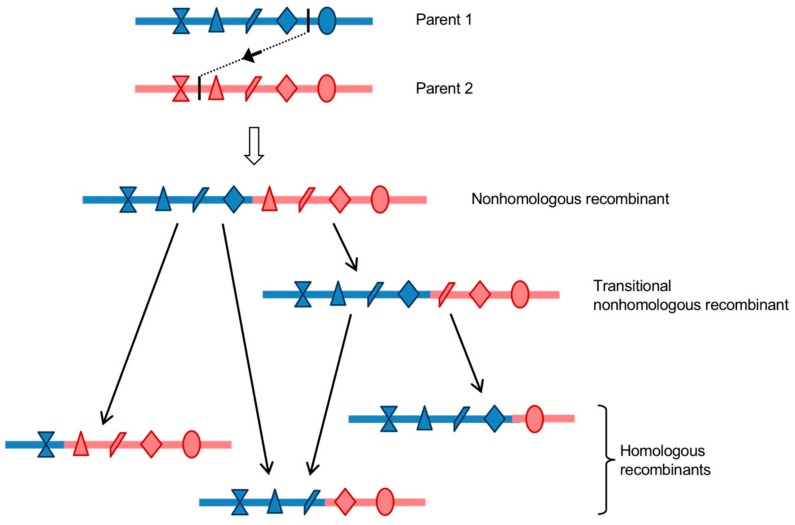
Model of generation of homologous recombinants from a nonhomologous one. A nonhomologous recombinant genome, displaying a duplication of homologous sequences around the recombination site, is produced by a replicative or nonreplicative recombination mechanism. Icons represent differences between the two homologous parental genomes. During the following replication cycles, the nonhomologous recombinant genome can generate a series of homologous recombinants through one or more rearrangement event(s). Recombination and rearrangement may take place within the initial co-infected (or co-transfected) cell, or in a different cell following re-infection, supposing that the nonhomologous recombinant can be encapsidated [189].

Most of the isolated nonhomologous recombinant genomes displayed inserted sequences of a variable number of additional nucleotides, which usually created duplications of homologous parental sequences on each site of the recombination site, and could reach a length of around 570 nucleotides in the 5’ UTR [77] and 148 codons in the non-structural region [187]. However, none of the natural recombinant EVs reported to date, including cVDPVs, exhibit clear signs of genomic duplications. Studies of some of the nonhomologous recombinants with insertions that were obtained with the recombination cellular systems previously described showed that they had growth and fitness disadvantages as compared to parental and homologous recombinant strains [77,121]. These nonhomologous recombinants would then only be present transiently in infected cells and organisms. Analyses of the in vitro and in vivo evolution of the duplicated sequences that were located in the non-structural region or in the 5’ UTR showed that they were frequently progressively deleted by genomic rearrangement following passaging in cells or animals, resulting in homologous recombinants [77,121,122]. These genomic rearrangement events are likely replicative, given that a functional polymerase is available to the virus. Indeed, it has been shown, in various positive-strand RNA viruses, that copy-choice recombination is capable of precisely removing genomic duplications with high efficiency [35,163,192,193]. Moreover, genomic rearrangement events are thought to be largely responsible for the formation of defective interfering particles (DIs), which are truncated forms of viral genomes that accumulate during replication of RNA viruses [126,194,195]. A Sindbis virus artificially modified in its polymerase was found to overproduce DIs, supporting the hypothesis of the implication of a replicative recombination mechanism [194]. As mentioned earlier in this review, the frequency of replicative recombination is positively correlated to the percentage of sequence identity between the two parental sequences [79,121,149,164,165,196,197]. Consistent with these reports, genomic rearrangements were found to occur faster when the sequence identity that was shared by the parental duplicated sequences increased [77]. Thus, first-generation nonhomologous intertypic recombinants undergo maturation through one or more subsequent genomic rearrangement events, which lead to the emergence of fitter homologous recombinants. A single nonhomologous recombinant was shown to be able to generate several different homologous recombinant genomes [121,122]. Nonhomologous recombinants could then be considered as precursors of the diversity of homologous recombinants genomes (Figure 6). In the case of intratypic recombination, the homologous recombinant genomes, which accounted for the majority of the isolated recombinants, might have been generated through a single precise recombination event, or may be the result of a faster evolution of nonhomologous recombinants promoted by the sequence identity level between the parental duplicated sequences.

To conclude, these recent experimental studies of recombination in EVs led to the elaboration of a new model for the generation of homologous recombinant EV genomes. Homologous recombinant EVs that are observed in nature could have directly arisen through replicative homologous recombination, according to the previously proposed copy-choice model, or as the result of successive nonhomologous recombination events. In the latter scenario, initial nonhomologous recombinants, which could have been generated by either replicative or nonreplicative recombination, would then evolve into homologous recombinants over one or more subsequent replicative recombination events. Nonhomologous recombinant genomes would function as precursors and recombination intermediates in a multi-step process that leads to the emergence of a diversity of homologous recombinants (Figure 6). This model could be generalized to other positive-strand RNA viruses, as suggested for HCV [28].

## 6. Recombination in Enteroviruses, Experimental Evidences of a Modular Evolutionary Process

### 6.1. Location of Recombination Hotspots

Another common feature of the viable recombinant genomes that were isolated from the recombination cellular systems is that recombination sites appeared not to be randomly distributed within the targeted genomic region, instead being located in recombination “hotspot” regions. Experimental studies of intertypic RNA recombination between PV and EV genomes enabled defining six recombination hotspots throughout the EV genome: three in the 5’ UTR and three in the non-structural region [77,121,122]. The distribution of recombination sites seemed to depend on the type of recombinant. Nonhomologous recombination sites were exclusively found in the hotspot sequences, whereas homologous recombination sites could also be found elsewhere in the targeted region. The recombination cellular systems were designed to analyze early but viable products of recombination. Thus, the features of the isolated recombinant genomes, including the recombination sites location, were the result of combined mechanistic and viability constraints. Importantly, a first study by deep sequencing of both viable and defective homologous recombinants produced by intratypic recombination in PV type 1 (PV1) identified recombination sites located all along the genome [153]. These results suggest that the recombination hotspots previously described might correspond to regions where recombination is more likely to produce viable genomes rather than sequences where recombination is mechanically favored, even though this last hypothesis cannot be fully excluded.

**Figure 7:**
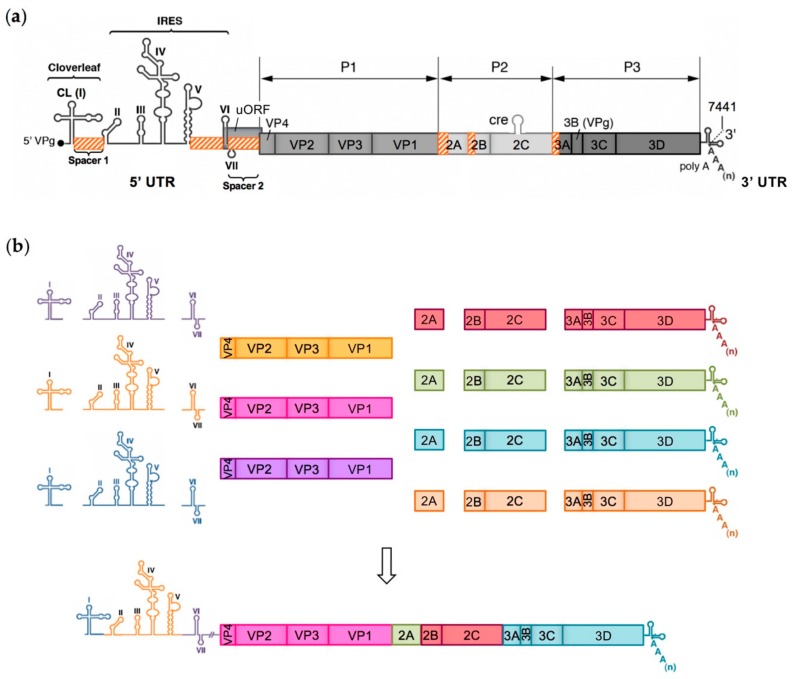
Model of modular evolution of species C enteroviruses. (**a**) Schematic representation of the enterovirus genomic RNA molecule. Experimental studies of genetic exchanges between poliovirus and enteroviruses led to the identification of six putative intertypic recombination hotspots, indicated by hatched orange rectangles [77,121,122]. (**b**) Modular recombination process. Each enterovirus species would exist as a pool of genetic material containing a finite set of P1 regions defining different types and a swarm of nonstructural and untranslated regions, divided in functional recombination modules and evolving independently. Each new enterovirus lineage can be considered as a new association of compatible recombination module.

Figure 7a presents the location of the six recombination hotspots. Three recombination hotspots were identified in the 5’ UTR: spacer 1 between the CL and the IRES, the linker sequence between IRES domains dV and dVI, and the dVII-spacer 2 region linking the IRES to the ppORF [77]. Three other recombination hotspots were identified in the non-structural region, each one being predominantly constituted by nonhomologous recombination sites and located at the junction between two viral genes: VP1-2A, 2A-2B, and 2C-3A [121,122]. Interestingly, the existence and location of these recombination hotspots appeared not to depend on the parental strains used as RNA partners, and may thus be considered as a general feature of RNA recombination between PV and EV genomes. However, it is important to emphasize that these recombination hotspots were identified in EV genomes that were produced in artificial recombination cellular systems, where environmental conditions and constraints are obviously very different from those of an in vivo infection. Yet, interestingly, the recombination hotspots identified in the in vitro selected recombinants correlated quite well with those of the natural cVDPVs lineages described so far. Indeed, among the 26 genomes of natural cVDPV lineages described to date, which were recombinant between OPV and non-PV EV-C sequences, 17 displayed a recombination site that was located in one of the three hotspots experimentally identified in the non-structural region (Figure 3). Eight recombination sites were located in the VP1-2A hotspot, seven in the 2A-2B hotspot, and two in the 2C-3A hotspot [65,66,67,68,69,70,71,72,73]. The nine other cVDPV lineages had recombination sites that were located in other sites of the nonstructural region, in particular in the 2C gene. Moreover, five of the cVDPVs displayed a second recombination site in the non-structural region, at the end of the 3C or 3D genes. In addition, among the 12 natural recombinant type 2 cVDPV lineages with non-PV EV 5′ UTR sequences, ten showed recombination sites in the dVII-spacer 2 region and two in the linker sequence between domains dV and dVI (Figure 3) [70,71,72,73]. Recombination in spacer 1 has not been described so far in natural cVDPVs.

Given that the recombinants that were isolated from the in vitro recombination systems were viable viruses replicating in cultured cells, it was also interesting to investigate their ability to replicate in vivo and their pathogenicity following the inoculation of transgenic homozygous PVR-Tg21 mice, which constitutively express the human PV cellular receptor CD155 [198,199]. In the case of recombination in the 5′ UTR between an EV 5′ partner from 5′ UTR group I (EV-C or EV-D) and a PV2 3′ partner, homologous recombinants with recombination sites located at the three identified hotspots appeared to be as neurovirulent as the PV2 partner [77]. Homologous as well as nonhomologous recombinant viruses between a PV2 5′ partner and a CV-A17 3′ partner with recombination sites located in VP1-2A or 2A-2B hotspots were also found to replicate and be pathogenic in mice [76,122].

### 6.2. Modular Recombination Process

The existence of recombination hotspots in the 5’ UTR and the region encoding nonstructural EV proteins was demonstrated in intertypic recombinants that were obtained from recombination cellular systems [77,121,122]. These recombination hotspots flank genomic sequences with very low rates of recombination, thus defining “recombination modules”. As previously mentioned, the hotspot location, and so the recombination module boundaries, likely result from the selection forces acting on and preserving viral functions. The modules could then be considered as functional recombination modules. The 5’ UTR and the non-structural region of EVs appeared to be composed of three recombination modules each: the non-coding regions of the CL, of the IRES domains dII to dV and of the domain dVI, and the regions encoding viral proteins 2A, 2BC, and 3ABCD (Figure 7). These genomic recombination modules are exchanged through RNA recombination occurring at the defined hotspots. Thus, intertypic EV recombination appeared to be a modular process, during which successive recombination events involving hotspots would lead to the construction of mosaic EV genomes.

In conclusion, the different recent studies that are presented in this review, which investigated the genetic exchanges between a panel of EV genomes in cellular systems, enabled experimentally demonstrating the theoretical model of EV evolution through modular recombination that had been proposed from phylogenetic studies of EV strains (see §2.4). These studies provided experimental evidence supporting phylogenetic data that EV genomes should be considered as combinations of genomic fragments, or recombination modules. These recombination modules would evolve independently and combine through RNA recombination during co-infections. The possible combinations of recombination modules generating viable EV genomes could be determined by preferred mutual functional compatibility, which was shown to be strikingly conserved between co-circulating EVs of the same species [78,97]. A recent study of the patterns of intertypic recombination of Sabin PVs demonstrated that the intrinsically higher/lower relative fitness of the recombination modules also played a significant role in their acquisition/loss by recombination [117]. Furthermore, these experimental studies enabled defining the boundaries of the recombination modules, at least in the context of in vitro PV/PV and PV/non-PV recombination. Some of the recombination modules that were identified correlated with known functional units, like the CL, the IRES and uORF in the 5′ UTR, and the 2A and 2BC proteins in the non-structural region.

## 7. Concluding Remarks

Despite the importance of RNA recombination in the evolution of EVs [61,106,111], as well as other important positive-strand RNA virus pathogens [91,200,201], the underlying mechanism(s) by which recombinants arise remains relatively poorly understood. Recent experimental studies that relied on recombination cellular systems mimicking natural genetic exchanges between EVs provided new insights into the molecular mechanisms of recombination in EVs, and enabled defining a new model of EV evolution through recombination [77,121,122,187,188]. The obtained data suggest that homologous intertypic recombinant EVs observed in nature are the final products of a multi-step process during which precursor nonhomologous recombinant genomes are generated through an initial inter-genomic recombination event and can then create a diversity of homologous recombinant genomes over one or more subsequent genomic rearrangement(s). Two main nonexclusive mechanisms of RNA recombination exist in EVs: a replicative copy-choice mechanism and a nonreplicative breakage-ligation mechanism. However, their relative contribution to the composition of the recombinant swarm remains unclear. In the recombination cellular systems that are reviewed here, co-transfecting cells either with two defective RNA partners or with a defective and a replicable RNA partners produced viable recombinant progenies with similar genomic features, which suggests a common intertypic RNA recombination process. This process would involve a first recombination step either replicative or nonreplicative, mostly producing nonhomologous recombinant genomes, followed by rapid evolution through replicative recombination, leading to the excision of the duplicated regions. Nonreplicative recombination and nonhomologous recombinant production seem to be favored in these recombination cellular systems in which RNA partners are nonreplicable and cells are co-transfected with great amounts of RNA molecules, which likely activates cellular RNA degradation pathways that are supposed to be involved in the nonreplicative breakage-ligation mechanism. Thus, it remains to determine whether this mechanism is still relevant in the context of natural infection and the recombination of functional genomes.

Moreover, these experimental studies highlighted a novel aspect of the organization of the EV genome. The genome of EVs could be defined as a combination of genomic modules that can be exchanged through RNA recombination. These results provided the first experimental evidence that supported the theoretical model of EV modular evolution previously elaborated from phylogenetic studies [106,111]. The recombination module boundaries were experimentally defined in the context of intertypic PV/PV and PV/non-PV recombination, which is implicated in the emergence of cVDPVs [77,121,122]. A good correlation was observed between the recombination hotspots that were experimentally located and the features of the natural cVDPV genomes described to date, validating the relevance of these recombination cellular systems mimicking natural genetic exchanges between EVs. It is important to note that this modular process might not apply to intratypic recombination, or that the location of the functional recombination module boundaries may be different, given that the structural and functional compatibility of recombining sequences is much higher in the context of intratypic recombination. In addition, as most of the results supporting the concept of modular evolution were obtained from recombination experiments that involved PV, it requires further investigation in non-PV EVs. It remains, in particular, to determine whether the location of the intertypic recombination hotspots could be generalized to all the EVs. A recent large-scale analysis of intertypic recombination patterns in human EV-A, EV-B, and EV-C genomes detected two of the three recombination hotspots that were experimentally identified in the 5′ UTR: the linker sequence between IRES domains dV and dVI, the latter containing the initiation codon of the uORF, and the dVII-spacer 2 region linking the IRES to the ppORF [77,108]. It is worth noting that the dVII-spacer 2 region, which was found to be the most prominent recombination hotspot in both experimental and phylogenetic studies, is located within the uORF harbored by the majority of EV-A and EV-B genomes and around half the EV-C genomes, which suggests a high tolerance of the single uORF protein to sequence alterations. Regarding the non-structural region, Nikolaidis et al. identified the P2 region, and, in particular, the 2A gene, as a preferential recombination region in circulating EV-B and EV-C genomes [108]. This observation is compatible with the existence of recombination hotspots that are located at VP1-2A and 2A-2B junctions that would lead to the generation of nonhomologous recombinant genomes further evolving into homologous recombinants with recombination sites in the 2A gene, as postulated by the proposed multi-step modular recombination model. In addition, the location of these two experimentally identified recombination hotspots is consistent with the high flexibility of EV 2A protein previously reported [202,203]. However, while the experimental systems of PV/EV-C recombination in the non-structural region enabled detecting only one additional intertypic recombination hotspot, at the 2C-3A junction (Figure 7), phylogenetic studies on circulating human EV genomes reported a more even distribution of recombination sites across the whole P2 and P3 regions [86,108,109,110,111]. One hypothesis that might explain this discrepancy is that the location of the non-structural recombination hotspots, and so the boundaries of the recombination modules constituting the EV non-structural region, is type- or genogroup-specific. Type-specific recombination modules were indeed recently identified in the non-structural region of Sabin 1, 2, and 3 PV genomes [117]. Furthermore, the recombination module boundaries could also depend on the nature of both recombination partners, as some asymmetries in reciprocal recombination were reported [74], and that certain species are thought to be more tolerant to recombination than others [108,111].

The structural and functional constraints that limit the exchanges of functional recombination modules also require further evaluation. For instance, recombination within the coding regions of human EV genomes has been thought to only occur among members of a given species [111]. However, interspecies recombination events in the non-structural region of the genome were found to have led to the emergence of non-human primate EV isolates within the EV-A and EV-B species [204]. Furthermore, genomes of porcine EV strains from the EV-G species were recently shown to have acquired a functional gene sequence from a Torovirus, member of the *Coronaviridae* family, by recombination at the VP1-2A junction [45,46]. These data suggest that the EV genome plasticity might be even higher than previously thought, at least in certain EVs infecting non-human animals.

Finally, the concept of genome evolution through exchanges of recombination modules might apply to other RNA virus families. Indeed, similar recombination models have been suggested for viruses from the *Flaviviridae* family [205], for coronaviruses [206] and BMV [207]. At a larger scale, the study of the macroevolution of invertebrate RNA viruses revealed patterns of modular genome evolution through widespread recombination among structural and non-structural genomic regions, which leads to the acquisition, removal, and exchanges of functional units over long evolutionary timescales [208]. Thus, to a different extent, modular genome evolution might be considered to be a common feature of RNA viruses.
